# Clinical, evolution and therapeutical considerations upon a case of fibrodysplasia ossificans progressiva (FOP)

**Published:** 2013-12-25

**Authors:** O Rogoveanu, R Traistaru, CT Streba, Z Stoica, R Popescu

**Affiliations:** Craiova University of Medicine and Pharmacy, Romania

**Keywords:** fibrodysplasia ossificans progressive, medical rehabilitation, soft tissue calcifications, osseous deformities

## Abstract

Abstract

Fibrodysplasia ossificans progressiva (FOP) is an autosomal dominant genetic transmitted disease, with a rare incidence (1-2 cases/million persons) and it usually affects female patients. Its manifestations include acute pain episodes that tend to repeat, involving the soft tissue and the axial muscles with later appearance of ectopic bone tissue in ligaments, joints and tendons. In the great majority of times, the skeletal modifications are observed at birth but the first clinical symptoms occur at 2-4 years old. The clinical symptoms include pain and inflammation of the soft tissue, sometimes associated with fever and cutaneous erythema, joint symptoms – pain, stiffness most frequently concerning the scapular and pelvic girdle, bone malformations - short hallux, microdactilia, kyphoscoliosis, thorax malformations. The diagnosis is established based on the clinical symptoms and the imagistic investigations: CT, MRI – which indicate the joint modification and the ectopic bone tissue. Muscular biopsy is not indicated as it leads to new lesions in the already traumatized areas.

## Introduction

Fibrodysplasia ossificans progressiva (FOP) is an autosomal dominant genetic transmitted disease, with a rare incidence (1-2 cases/million persons) and it usually affects female patients [**1**–**3**].

Its manifestations include acute pain episodes that tend to repeat, involving the soft tissue and the axial muscles with later appearance of ectopic bone tissue in ligaments, joints and tendons [**1**,**4**,**5**].

In the great majority of times, the skeletal modifications are observed at birth but the first clinical symptoms occur at 2-4 years old [**6**,**7**].

Clinical symptoms include pain and inflammation of the soft tissue, sometimes associated with fever and cutaneous erythema, joint symptoms – pain, stiffness most frequently concerning the scapular and pelvic girdle, bone malformations- short hallux, microdactilia, kyphoscoliosis, thorax malformations [**1**, **7**–**10**].

 The diagnosis is established based on the clinical symptoms and the imagistic investigations: CT, MRI – which indicate the joint modification and the ectopic bone tissue [**5**,**7**,**11**]. Muscular biopsy is not indicated as it leads to new lesions in the already traumatized areas [**9**,**12**].

The disease has a negative evolution and a reserved prognosis due to the bone modification and the thorax muscular involvement that later triggers restrictive respiratory dysfunction [**1**–**13**,**9**–**13**]. There is no pathogenic treatment, only a symptomatic one, during the acute phase of the disease, the genetic therapy being the only perspective for solving these medical cases in the future [**10**–**14**]. 

## Clinical case presentation

**Anamnestic data**

We present the case of a 36-year-old patient, from rural environment, who was admitted to the Recovery Medicine Clinic of the Emergency County Hospital of Craiova, with physical asthenia, myalgia of the scapular and pelvic girdle, lumbar and cervical pain, static and walking dysfunction, functional disability concerning the elbow, scapulohumeral and coxo-femoral joints in relation to daily activity motor actions. The patient had been first diagnosed with FOP 10 years before during her first admission in the hospital. Personal data indicated that she had suffered a left ankle fracture that was later orthopedically treated, she had been diagnosed with essential scoliosis at the age of 7 and had a surgery intervention for submandibular cyst. At the age of 19 she was diagnosed with non–septic femoral head necrosis and by the age of 25 (May 2002) she suffered a falling trauma with a fracture of the bone structure between the left subscapular muscle and the third left rib posterior arch. 

The medical history of the patient showed numerous intramuscular and intravenous injection therapies, administration of NSAID and steroid anti–inflammatory drugs and also the falling trauma that could lead to FOP.

**Clinical examination**

The patient inspection revealed a deficient nutrition, underweight (35kg, 1.60 m thus a body mass index of 13.7). The face was myopathic, with maseterina muscles hypotrophy. The skin was pale, the thorax was deformed with scoliosis and we observed decreased respiratory movement amplitude concerning the left hemitorax during palpation. The patient accused anorexia but there were no objective deglutition modifications.

**Recovery medicine examination**

**Fig. 1 a, b:**
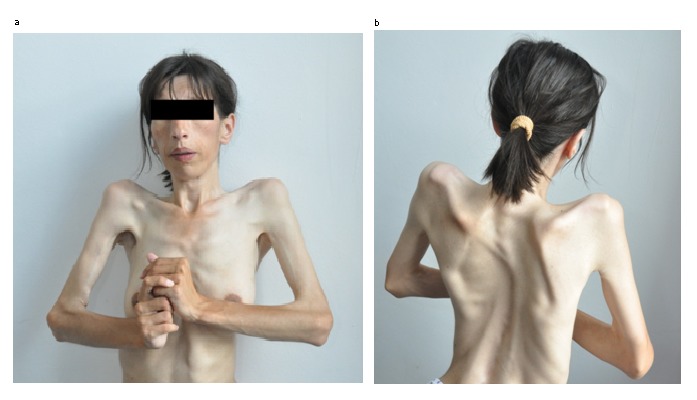
Front and back views. Dorsal dextroconvex ‚ “S”–shaped scoliosis and deformed thorax. Fixed retraction of elbow flexion muscles (biceps, brachial, brachioradial) with bilateral elbow ankylosis in 90 degrees flexum

During a detailed medical checkup we observed a general amyotrophy, limitation of mouth opening (3 cm) due to atrophy/constriction of maseterina muscles, pain and constriction of cervical muscles with limited mobility: IMS=10cm, IOP=0 cm, ITA=15 cm. We also noticed dorsal dextroconvex‚ “S” shaped scoliosis and deformed thorax (**Fig. 1 a,band 2**), I cirt=3,5cm, Schober index=1cm; retraction of pectoral muscles (with a 4,2cm hard, bone tissue accumulation at the medial third, across the anterior axillary line), also the deltoid, coracobrachial muscles with the fixation of scapulohumeral joints in an anatomical position and with the presence of hard bone consistency accumulations of 4/3cm at postero-lateral inferior third of the right forearm and at inferior third of right arm.

**Fig. 2:**
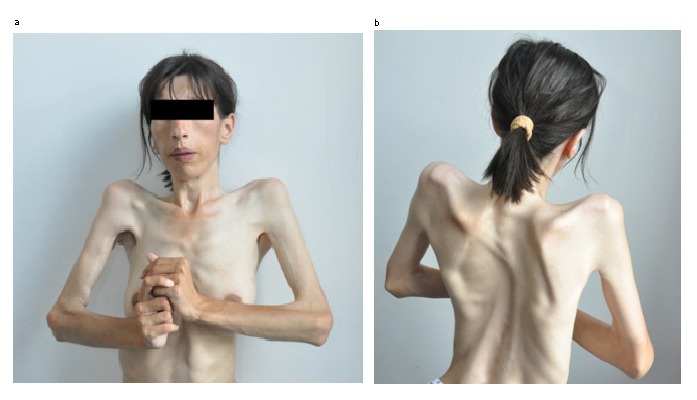
Lateral view. Lordosis, dextroconvex ‚ “S”–shaped scoliosis and deformed thorax. Fixed retraction of elbow flexion muscles (biceps, brachial, brachioradial) with bilateral elbow ankylosis in 90 degrees flexum

**Fig. 3 a,b:**
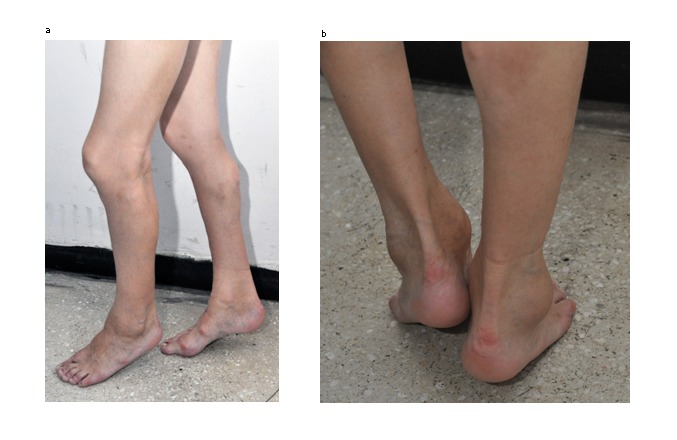
Soft tissue calcifications

Regarding both upper limbs we observed a fixed retraction of the elbow flexion muscles (biceps, brachial, brachioradial) with bilateral elbow ankylosis in 90 degrees flexum (**Fig. 1-3**). Concerning the inferior limbs, we noticed painful, contracted gluteus, hip adductor muscles with mobility A/P: F=30 degrees, RI=0, RE=0, Abd=0; retraction of left isckial left muscles with fixed flexum of the left knee=15 degrees and the presence of bone consistency accumulations of 7/6 cm at the right sural triceps muscle at the medial half. Osteotendinous rotulian and Achilles reflexes were present but diminished. The patient could walk without any support in small dangled steps.

**Laboratory and imaging exams**

From the laboratory data, we retained Hb 10.4 mg%, ESR, glucose, GOT, GPT, urea, creatinine, urine examination summary, CRP, fibrinogen, CPK, LDH within physiological limits.

EMG - the collection of tibialis anterior muscles – poor interference paths, when collecting from the thenar eminence - trails with giant potential. Ventilatory function tests showed a restrictive type ventilatory dysfunction without significant changes in blood gases.

**Fig. 4:**
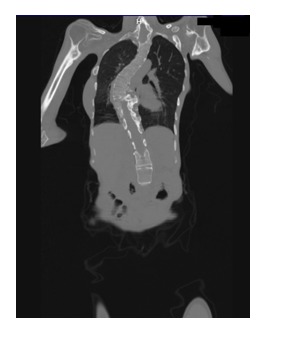
CT aspect. Dextro-converse scoliosis; bone thorax deformation. Pulmonary fibrosis.

**Fig. 5:**
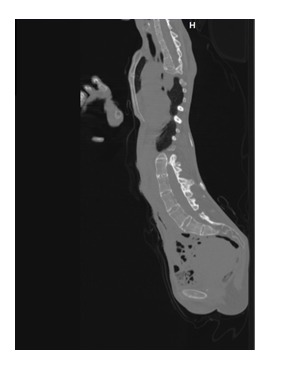
CT aspect. Marked kyphoscoliosis deformities. Lateral view.

**Fig. 6 a,b:**
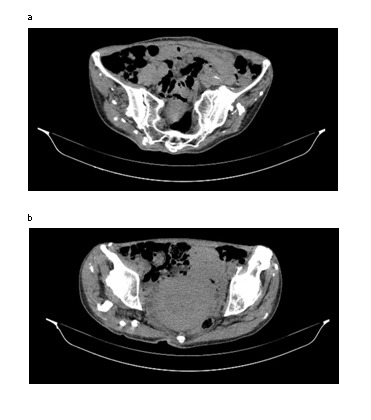
CT aspect. Calcifications of the gluteal muscles and at the trochanterian insertion of the left psoas muscle

**Fig. 7:**
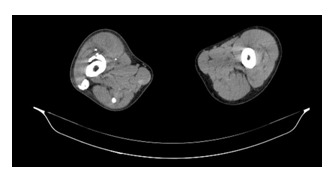
Calcifications of femoral quadriceps muscles

**Fig. 8:**
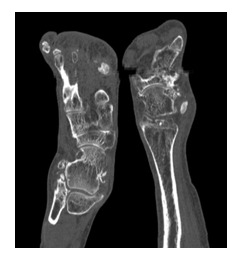
CT aspect. Soft tissue calcifications in the lateral region and deformation of the left thallus

**Fig. 9:**
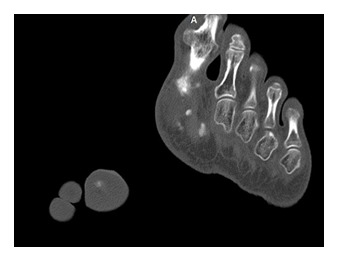
CT aspect. Deformations of the hallucal bone

Cervical spine radiograph revealed bone block C2–C3. Chest radiography and CT were performed which revealed soft tissue lesions of pulmonary fibrosis (**Fig. 4 and 5**) and calcification of the periarticular soft tissue extended to the lower limbs, glenohumeral joints and muscles round the backbone (**Fig. 4 - 9**), spine with marked kyphoscoliosis deformities which contributed to increased restrictive ventilatory dysfunction.

## Results and discussions

The patient was under our supervision for the past 13 years (2000–2013) and followed four annual treatment courses of complex rehabilitation in the Clinic of Physical Medicine and Rehabilitation of the Medical Hospital No. 1 Craiova.

Given the advanced clinical and functional stage and of morbid complications, we had as the first objective the influencing of algal symptoms (indomethacin 50mg 1cp x 3/day, celecoxibum 100mg 1cp x 2/day, analgesic electrotherapy, sedative massage, cryotherapy 4 times a day, 10 minutes each and bio feedback relaxation techniques).

To combat contraction/ muscle retraction we administered tolperisone chlorate 100mg/day, gabapentin 100mg/day, also making superficial mild heat massage-passive muscle stretching and PNF-hold-relax techniques. In order to resolve positioning anomalies and increased joint mobility we have recommended posture, morning, passive physical therapy consisting of passive motion and daily active physical therapy in two sessions of 15 minutes each.

To maintain and improve the respiratory function we performed diaphragmatic breathing exercises, deep counter-resistance inspires, asuplisation of the spine, postural reeducation and classical active physiotherapy.

In order to combat anemia and fatigue we used iron preparations, vitamins; we set a high protein and high-caloric diet. Measurements of rehabilitation to usual gestures and social reinsertion required education, psychotherapy and occupational therapy consisting in the performance of manual tasks.

The patient was diagnosed based on clinical data and imaging with advanced FOP, restrictive respiratory dysfunction and secondary anemia.

Regarding pain assessed by VAS (score range 0-10), we observed an alleviation consecutive to respiratory cures of about 3–4 points.

Movement amplitude (AM articular testing): we did not register significant increases in AM, especially in SH and elbow joints that would allow self-service of the patient.

Motor skills were assessed by using the SAM scale with scores from 0 to 11, the patient presenting an average SAM score of 4, without significant improvements during therapy.

The nutritional status improved gradually, resulting in a decrease of anorexia, with a record of 3–4 kg weight gain consecutive to treatment (probably by improving mental tone), resulting in an improvement in anemia.

The evolution of the weight curve underwent a marked improvement, this involving an improvement of all blood constants objectified by the reduction of the anemia. The patient's mental status was also improved, this being a beneficial effect on the progression of states of fatigue and general condition.

The evolution was progressive, with functional complications and disabilities consisting in fractures. Ad vitam prognosis was good, but the functional prognosis was still reserved.

The diagnosis was delayed in this patient; there were a series of events that precipitated its evolution to advanced stages during the course of the disease. Numerous injectable treatments, both intramuscularly and intravenously over the course of admissions, muscle trauma and a history of repeated interventions are among the main factors aggravating the patient's history.

A determining role was played by the multidisciplinary recovery performed by a physician, physical therapist and psychotherapist, each with specific methods obtaining result that acted synergistically on disease progression [**1**–**3**,**15**–**17**].

## Conclusions

Fibrodysplasia ossificans progressiva or progressive myositis is a disease of invaliding progressive congenital disabilities. Early diagnosis (anamnestic, clinical emphasizing malformations or imaging – radiological) is a first important step in the rehabilitation program. Aggravating factors such as trauma to the muscle or medical maneuvers (intramuscular or intravenous injections, muscle injuries or surgery) should be known to the medical staff and avoided as much as possible. Recovering patients with FOP is a complex and laborious medical act which should be initiated as early as possible, by a multidisciplinary team (physician, physical therapist, and psychotherapist); kinetic methods are essential, being a mandatory part of the rehabilitation program.
